# Creutzfeldt-Jacob disease presenting as severe depression: a case report

**DOI:** 10.1186/1757-1626-2-122

**Published:** 2009-02-04

**Authors:** Ethan Cumbler, Kristin Furfari, Jeannette Guerrasio

**Affiliations:** 1 Division of General Internal Medicine, Department of Medicine, University of Colorado Denver School of Medicine, Hospital Medicine Service, F782, Colorado, USA

## Abstract

**Background:**

An 81 year old female presented with altered mental status after new onset of severe depression and suicidal ideation with recent psychiatric hospitalization.

**Case presentation:**

Key clinical features included muscle rigidity, prominent startle reflex, and rapidly progressing cognitive decline. Initial working hypothesis was serotonin syndrome or neuroleptic malignant syndrome but continued deterioration after medication removal prompted evaluation for alternative etiology. Work-up revealed elevated 14-3-3 CSF protein which suggested the prion disorder which was confirmed on post-mortem examination of brain tissue.

**Conclusion:**

While the degree of depression was unusually severe, the case highlights the behavioral and psychiatric manifestations which frequently accompany Creutzfeldt-Jacob disease.

## Case study

81 y/o previously highly functioning white female was admitted for altered mental status. Recent history was significant for development of new severe depression with suicidal ideation, attributed to psychosocial stressors, one month prior to presentation. During an initial 2.5 week inpatient psychiatric hospitalization, she was treated with venlafaxine and quetiapine with apparent initial response. Soon after discharge from the psychiatric hospital the patient was readmitted with rapidly worsening confusion, decreased p.o. intake, and "catatonic" behavior.

Past history was significant for waldenstroms macroglobulinemia. She lived alone in rural Colorado and engaged in extensive international travel most recently to scuba dive off the Great Barrier Reef at the age of 80.

Physical exam was significant for non-oriented female with flat affect who followed simple commands but without spontaneous speech. Her musculoskeletal exam revealed 5/5 strength with lower extremity rigidity and a startle response to touch.

Initial hypothesis was an adverse drug event such as neuroleptic malignant syndrome or serotonin syndrome. Differential diagnosis included catatonic depression, central nervous system infection, hyperviscosity, or paraneoplastic syndrome.

Selected testing performed included brain MRI which demonstrated cerebral atrophy and moderate to marked white matter abnormality, electroencephalogram with moderate diffuse background slowing and shifting lateral predominance, but without classic periodic sharp waves, and a lumbar puncture with protein of 143 mg/dl (normal range 15–45 mg/dl) and elevated 14-3-3 protein. Over her 16 day hospitalization the patient demonstrated a progressive loss of ability to follow commands, posturing, and transient focal left sided facial droop and weakness. She was transferred to hospice and died 5 days later. Autopsy, with western blot and PCR on brain tissue, confirmed sporadic Creutzfeldt-Jacob Disease.

## Discussion

Creutzfeldt-Jacob Disease (CJD) is a rapidly progressive spongiform neurodegenerative disorder caused by prion proteins. The case rate is approximately 1 per million with an average age of onset of 60.7 years [1,2]. The latency period can last years, but time from onset of symptoms to death is typically less than a year [2]. Eighty five percent of cases are due to spontaneous protein transformation or somatic gene mutations while direct infectious transmission and familial mutations account for the remaining cases. Commonly presenting as a rapidly progressive dementia, CJD is characterized by cognitive changes, gait disturbance, akinetic mutism, and myoclonis. Behavioral changes, seen in 30% of patients at the onset and 55% of later disease stages, are common [3].

Initial presentation as severe depression with suicidal ideation prompting inpatient psychiatric hospitalization is unusual. A review of 126 cases of CJD over a 25 year period found six who were hospitalized for symptoms of depression during the course of their disease [4]. Psychiatric symptoms are frequently treated with antipsychotics, anxiolytics and antidepressants but often have limited and temporary benefit.^4 ^Antidepressants were initiated in 22 of the 126 reviewed CJD cases with only one case documenting benefit.

Rigidity and altered mental status occurring with initiation of psychiatric medications may lead to initial consideration of serotonin syndrome or neuroleptic malignant syndrome. However, in CJD elevated blood pressure and fever would not be expected and symptoms will not improve with removal of medications.

This case emphasizes the need to consider organic encephalopathy in the differential diagnosis of an elderly patient without prior psychiatric history who presents with new-onset severe depression. When the clinical picture contains motor symptoms such as rigidity or exaggerated startle response along with catatonic features, CJD should be entertained, particularly if cognitive function quickly declines and psychiatric symptoms fail to respond to therapy. Unfortunately, there is no effective treatment for this uniformly lethal disorder but early consideration and timely diagnosis can allow appropriate palliation and reduce the stress caused by uncertain etiology on family and caregivers.

## Family perspective

Virtually overnight, my vital, vigorous, feisty mother grew brittle, unsteady, incontinent and deeply depressed – during the last four months of her life, she repeatedly stated to me and others that she felt she was losing her mind. (An accurate assessment in hindsight; one to which we should have given more weight, since she was trained as a nurse and was the wife of a doctor, closely engaged with the medical profession for 64 years) This was a shocking change in our mother, since she was previously mentally and physically active; driving, volunteering as an R.N. for the Red Cross, travelling abroad, and avidly engaging the world around her.

Initially, many of her symptoms were dismissed or minimally explained – mobility/bladder control issues as age-related, depression as situational, rigidity/affect flatness as drug reaction, etc. It was frightening. Her decline – physical, cognitive and emotional – was dramatic. She lost control of thought processes and mood, was increasingly unstable on her feet, and her personality faded. Most disturbing was the seeming inability of medical professionals, until admission to University of Colorado Hospital, to fully appreciate the precipitous and pronounced changes we were observing that seemed to us to be atypical of age-related decline, different from situational depression, and certainly more long lasting than a possible drug reaction.

These early explanations were, to family and friends, improbable, and our frustration grew as every day we lost another part of the woman who had filled up our lives with exuberance, curiosity and passion. We felt there was a disconnect in the medical analysis, looking at a seemingly random menu of diagnoses ranging from psychiatric, to geriatric, to urologic, to neurologic. An earlier consideration of the possibility of Prion disease would have given us a rational, and therefore at least understandable, explanation for her decline and death.

## Consent

"Written informed consent was obtained from the deceased patient's daughter for publication of this case report. A copy of the written consent is available for review by the Editor-in-Chief of this journal."

## Competing interests

The authors declare that they have no competing interests.

## Authors' contributions

EC reviewed the patient's chart and prepared the case study for publication. KF and GC performed literature search and review of published data and were major contributors to the writing of the manuscript. All authors read and approved the final manuscript.

The patient's daughter prepared the family perspective portion of the manuscript and has reviewed the case study and discussion.
